# Asymmetric synthesis using chiral-encoded metal

**DOI:** 10.1038/ncomms12678

**Published:** 2016-08-26

**Authors:** Thittaya Yutthalekha, Chularat Wattanakit, Veronique Lapeyre, Somkiat Nokbin, Chompunuch Warakulwit, Jumras Limtrakul, Alexander Kuhn

**Affiliations:** 1 Univ. Bordeaux, CNRS UMR 5255, Bordeaux INP, ENSCBP, 16 Avenue Pey Berland, 33607 Pessac, France; 2 Department of Chemistry, Faculty of Science, Kasetsart University, Bangkok 10900, Thailand; 3 NANOTEC Center for Nanoscale Materials Design for Green Nanotechnology, Faculty of Science, Kasetsart University, Bangkok 10900, Thailand; 4 Department of Chemical and Biomolecular Engineering, School of Energy Science and Engineering, Vidyasirimedhi Institute of Science and Technology, Rayong 21210, Thailand; 5 Department of Materials Science and Engineering, School of Molecular Science and Engineering, Vidyasirimedhi Institute of Science and Technology, Rayong 21210, Thailand

## Abstract

The synthesis of chiral compounds is of crucial importance in many areas of society and science, including medicine, biology, chemistry, biotechnology and agriculture. Thus, there is a fundamental interest in developing new approaches for the selective production of enantiomers. Here we report the use of mesoporous metal structures with encoded geometric chiral information for inducing asymmetry in the electrochemical synthesis of mandelic acid as a model molecule. The chiral-encoded mesoporous metal, obtained by the electrochemical reduction of platinum salts in the presence of a liquid crystal phase and the chiral template molecule, perfectly retains the chiral information after removal of the template. Starting from a prochiral compound we demonstrate enantiomeric excess of the (*R*)-enantiomer when using (*R*)-imprinted electrodes and vice versa for the (*S*)-imprinted ones. Moreover, changing the amount of chiral cavities in the material allows tuning the enantioselectivity.

## Abstract (web summary)

Chiral compounds are extremely important as they can be used in medicine, agriculture and biotechnology. Here, Kuhn and co-workers use a mesoporous metal structure encoded with chiral information in order to induce asymmetry in electrochemical synthesis of mandelic acid.

## Graphical abstract

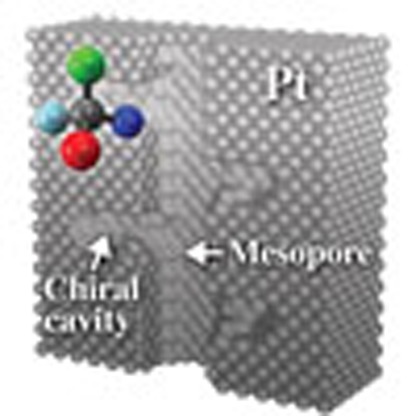

One of the fundamental and most fascinating aspects of life is the homochirality of biological molecules. Most chiral biological molecules exist in only one of two possible mirror-image forms. This has the important consequence that the biological and pharmaceutical activity of chiral molecules is directly related to their handedness, which means that the two different enantiomers of a molecule often exhibit markedly different effects. The selective synthesis of only one of the two mirror-image forms of a chiral molecule is therefore of major importance. In general, enantiomerically pure compounds can be obtained either by separation of a racemic mixture or chiral synthesis^1^,^2^. From a viewpoint of economic efficiency, enantioselective synthesis is more attractive than the separation of a racemic mixture, the latter one producing intrinsically half of the product as waste. Presently, the selective synthesis of enantiomerically pure compounds is a key process in modern chemistry and especially important in the pharmaceutical industry^1^,^3^,^4^.

Although great progress has been made with respect to homogeneous asymmetric catalysis based on transition metal complexes with various chiral ligands^5^,^6^, research to develop highly versatile, chiral, heterogeneous catalysts for asymmetric organic transformations has had only limited success^7^,^8^. Immobilization of homogeneous asymmetric catalysts or other, more or less complex, chiral molecules, such as, for example, cinchona alkaloids, on various types of solid support has been studied^9^,^10^,^11^,^12^, however, there might be limitations due to the stability of the catalysts. Immobilized chiral catalysts or chiral modifiers can detach from the solid support, resulting in weak or no surface chirality. Thus, the development of original and alternative methods to generate surfaces with chiral activity is still a great challenge for both, academics and industry and complementary approaches are explored at the moment^13^.

To favour the formation of one enantiomer with respect to the other, a chiral feature needs to be present in either the educt, the reagent, the catalyst or the local chemical environment. The impact of external physical factors on the enantioselective synthesis from achiral or racemic precursors without the use of chiral catalysts or chiral auxiliaries has also been proposed and is known as absolute asymmetric synthesis^14^. Different approaches have been investigated to influence the synthesis of chiral products, such as magnetic, electric and gravitational fields, as well as circularly polarized light^15^,^16^,^17^,^18^. Moreover, absolute asymmetric synthesis by means of a purely geometric approach, in which the macroscopic orientation of achiral precursors is controlled before the reaction, has also been suggested. Such a macroscopic orientation can be achieved, for example, through the natural molecular alignment of a prochiral precursor in single crystals^19^,^20^. It has been found that this geometric approach leads to higher enantioselectivity than most of the other absolute asymmetric synthetic schemes.

The spatial orientation of a prochiral precursor during the enantioselective synthesis can also be influenced by the presence of molecular chiral cavities in a solid or on its surface. With this respect, very interesting approaches have been put forward in the literature, mostly based on the concept of molecular imprinting of polymers (MIPs)^21^,^22^,^23^,^24^ or other materials^25^,^26^,^27^, and some authors could extend the concept even to metals^28^,^29^,^30^. As the matrices obtained by such an imprinting approach have cavities with a shape similar to the used template, this confined reaction space might be used to direct the product formation. There have been several reports on asymmetric synthesis using molecular imprinted polymers based on introducing a catalytic metal into the imprinted cavity in which the reaction is accomplished by metal catalysis, with the enantioselectivity being controlled by the chiral environment surrounding the metal^31^,^32^. This kind of polymer catalysts have been successfully applied for many systems such as the asymmetric reduction of prochiral ketones using Rh-containing MIPs^33^, asymmetric transfer hydrogenation of *o*-fluoroacetophenone with SiO_2_-supported Ru-MIPs^34^ and enantioselective ligand-exchange reactions using Pt-containing MIPs^35^. However, this pathway of molecular imprinting sometimes also suffers from disadvantages, such as difficult template removal or poor mass transfer. A reason for the usually observed low selectivity might be the relatively small active surface area, and therefore a small number of imprinted recognition sites, that are available when using flat surfaces. Therefore, a promising strategy to improve the amplitude of chiral recognition consists in using porous structures and in particular porous metals.

We have recently developed chiral-encoded mesoporous metals, which combine the benefits of a high active surface area with chiral discrimination and exhibit remarkable chiral recognition properties^36^. Moreover, such a chiral-imprinted mesoporous metal is not only able to discriminate the enantiomers of the imprinted chiral molecule but allows also recognition of other chiral molecules having a similar spatial configuration with respect to the imprinted one^37^. This recognition versatility is due to the geometric arrangement of the metal atoms in the imprinted cavities, which allows preferential interaction with the target molecule. Analogous to the geometric approach for absolute asymmetric synthesis, such chiral cavities might be used to control the spatial orientation of the prochiral precursor's reactive site due to the geometric nature of the chiral information of the cavities. Until now, such chiral-imprinted mesoporous metals have only been applied to the detection of enantiomers, but have not been tested so far for enantioselective synthesis.

Herein, we demonstrate the ability of chiral-encoded mesoporous metal structures to induce a certain degree of asymmetry in the synthesis of chiral products. In a proof-of-principle experiment, we investigated the enantioselective synthesis of mandelic acid (MA) based on the electrochemical reduction of phenylglyoxylic acid (PGA) on chiral mesoporous platinum that has been imprinted with MA. The prochiral starting molecule, which is interacting with the chiral metal cavity, is electrochemically reduced at the prochiral carbon atom, and the handedness of the resulting product can be influenced by the geometry of the chiral cavity. The degree of enantioselectivity can be tuned by changing the number of imprinted recognition sites.

## Results

### Synthesis of chiral-encoded mesoporous platinum surfaces

**Figure 1:**
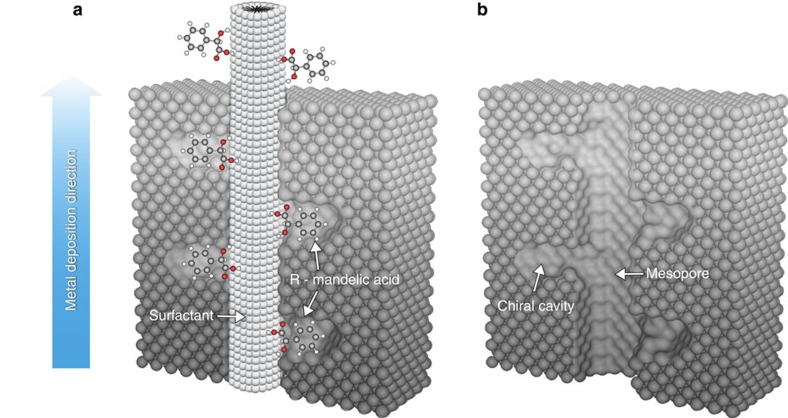
Schematic illustration of the synthesis of chiral-imprinted mesoporous platinum. (**a**) Electrodeposition of platinum (grey atoms) around the self-assembled structure of the liquid crystal phase (supramolecular column) and the adsorbed chiral template molecules (MA). (**b**) Model of the resulting structure after template removal, with chiral cavities decorating the inner wall of the mesoporous channels.

According to previous reports, the lyotropic liquid crystal of Brij 56 has a hexagonal (H_1_) structure, leading to the generation of mesopore channels. The interaction between the outer hydrophilic part of such a columnar Brij 56 liquid crystal structure and the COOH and OH groups of MA should allow adsorption of the latter one on the surface of the surfactant columns with a preferential molecular orientation (Fig. 1a). It is reasonable to assume that the chiral template is orientated with its hydrophilic part towards the equally hydrophilic outer surface of the surfactant columns, as schematically depicted in Fig. 1a. Obviously, other orientations are statistically also possible, but are much less likely in terms of interaction energies. We have calculated the difference of interaction energy between a situation where MA is orientated with its hydrophilic moiety towards the hydrophilic part of the Brij molecule and a configuration where the aromatic ring is pointing towards the Brij molecule. The latter orientation is 12.2 kcal mol^−1^ less stable, which is a very significant difference (Supplementary Discussion). The platinum salt, which is also present in the mixture, can be electrochemically reduced to produce a metal layer growing around the Brij 56 columns^38^. We assume that during the growth, the chiral templates, present at the periphery of the columns, can be engulfed by platinum, which wraps the bulky phenyl group and the asymmetric carbon atom (Fig. 1a). Removal of the surfactant and the chiral template (Fig. 1b) should therefore result in the formation of cavities of molecular dimensions in which the chiral information is encoded as a negative imprint (see Methods section for details of the preparation process). One might have the impression that the presence of low-coordinated metal atoms in these cavities is a rather unstable situation, however this depends very much on the type of metal. Indeed, attempts to produce comparable imprinted structures with soft metals such as gold failed because the metal is not able to encode molecular information due to the high surface mobility of the atoms. For platinum things are different as has already been demonstrated by the group of Attard^39^. They were able to show in a very different approach that when cutting a Pt monocrystal along a plane with a high Miller index, the generated surfaces exhibit chiral features due to the local arrangements of the individual atoms, which are stable over time even though they are constituted by low-coordinated metal atoms^40^.

### Characterization of chiral-encoded mesoporous platinum

**Figure 2:**
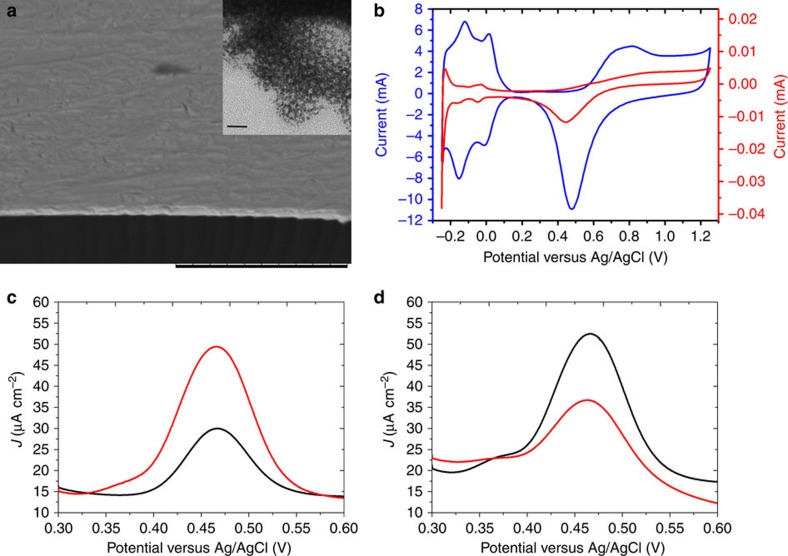
Characterization of chiral-imprinted mesoporous platinum. (**a**) Scanning electron microscopy image of a typical cross-section of the metal film. Scale bar, 10 μm. Inset:TEM image of the mesopores with a scale bar of 20 nm. (**b**) Cyclic voltammograms of flat-polished platinum (red) and a chiral-imprinted mesoporous platinum film (blue), recorded in 0.5 M H_2_SO_4_ at 100 mV s^−1^. (**c**,**d**) Differential pulse voltammograms in 4 mM D-DOPA (black) and L-DOPA (red) (50 mM HCl as supporting electrolyte) of chiral mesoporous platinum electrodes imprinted with (*R*)- and (*S*)-MA, respectively.

To use the chiral-encoded mesoporous platinum as a working electrode in the electrochemical synthesis study, the metal deposition has been carried out on gold-coated glass slides (1 cm^2^). The morphology of the platinum film was uniform over a wide range and its thickness was estimated to be ∼0.3 μm (Fig. 2a). The observed global morphology is similar to the one reported previously^36^, and from the inset one can clearly see the mesopores with a very uniform size. The active surface area of the porous platinum film was measured by cyclic voltammetry. As can be seen in Fig. 2b, the well-pronounced hydrogen adsorption and desorption peaks (−0.2 to +0.2 V), as well as the oxidation and reduction of Pt and PtO_*n*_, respectively (∼0.7 and ∼0.5 V) are significantly enhanced for the porous platinum (blue line) compared with a polished flat platinum electrode (red line). This translates the surface increase, which is expected for the mesoporous structure. According to the charge associated with the hydrogen adsorption and desorption processes, the electroactive surface areas of the chiral-imprinted mesoporous and flat platinum were determined to be 131 and 0.05 cm^2^, respectively (see Methods section). These values have to be renormalized with respect to the geometric surface areas of the two electrodes (1 and 0.03 cm^2^, respectively), which leads to roughness factors of 131 and 1.6 for the chiral-imprinted mesoporous and flat platinum electrodes, respectively. The high roughness factor value of the mesoporous electrode confirms the surface area enhancement, resulting from the porous nature of the electrodes. The accessible surface area can also be estimated with adsorbants that are bigger than hydrogen. Chemical entities that readily adsorb on a large variety of materials are polyoxometalates. We have studied the surface enhancement factor with such a bigger molecular probe (PMo_12_O_40_^3−^), well known for its chemisorption on platinum^41^ and other electrode materials^42^, and in this case roughness factor values of around 30 are measured (Supplementary Fig. 1 and Supplementary Discussion).

The chiral feature of the porous platinum film can be revealed by the recognition of 3,4-dihydroxyphenylalanine (DOPA) enantiomers, using differential pulse voltammetry^36^,^37^. The difference in amplitude of the D- and L-DOPA oxidation peaks (∼0.45 V) demonstrates the chiral recognition properties. The recognition versatility that results from the geometric arrangement of the metal atoms in the cavities allows electrodes, which have been imprinted with MA, to distinguish also DOPA enantiomers. (*R*)-MA-imprinted mesoporous platinum preferentially reacts with L-DOPA and vice versa for the (*S*)-MA-imprinted one (Fig. 2c,d). The observed behaviour is similar to the one reported in a previous study^37^ and therefore allows confirming the chiral feature of the electrodes. It is worth mentioning that if the imprinting is carried out without the surfactant almost no chiral discrimination is observed and therefore such electrodes have not been used for the subsequent asymmetric synthesis because the enantiomeric excess is expected to be neglectable.

### Enantioselective synthesis of MA

The electrochemical reduction of PGA to MA has been chosen for this study as a model reaction for the enantioselective synthesis on chiral-imprinted mesoporous platinum. A potential of −0.5 V versus Ag/AgCl has been applied to the working electrode, because the reduction of the prochiral C=O functional group in the PGA molecule starts to be significant around this potential value (Supplementary Fig. 2 and Supplementary Discussion). This rather modest negative potential has been chosen deliberately to avoid interference with the reduction of protons, which would produce hydrogen bubbles that might damage the mesopores. Owing to this relatively small driving force the currents during the electrosynthesis are only in the mA range (Supplementary Fig. 3).

**Figure 3:**
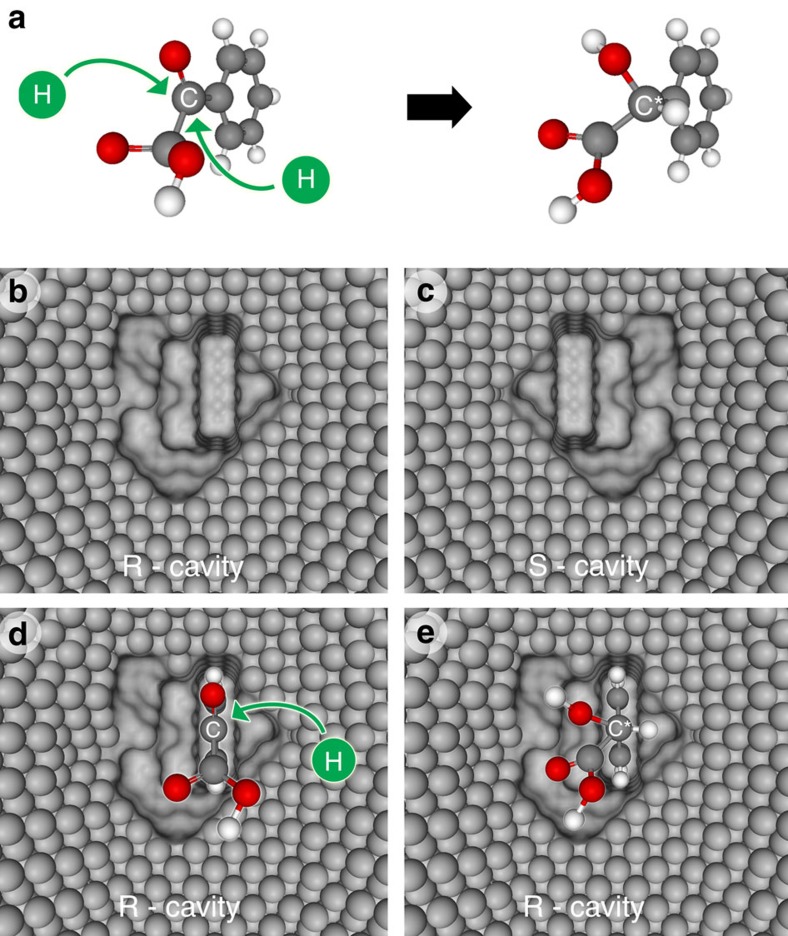
Illustration of the concept of enantioselectivity for the reduction of PGA to MA. (**a**) Schematic representation of the spatial configuration of the reactive prochiral carbon atom in PGA and the two possibilities to add hydrogen to form one or the other enantiomer. (**b**,**c**) Chiral cavities obtained by imprinting with (*R*)- and (*S*)-MA, respectively. (**d**) Interaction of PGA with the (*R*)-imprinted cavity, showing the influence of the geometry of the chiral metal cavity on the enantioselectivity of the product formation. (**e**) The resulting (*R*)-MA in the (*R*)-imprinted cavity.

The enantioselectivity in the electrochemical reduction of PGA (Fig. 3a) on chiral encoded mesoporous platinum is supposed to be due to the spatial orientation of the prochiral precursor. The chiral cavity produced via the imprinting process (Fig. 3b,c) provides an asymmetric environment, giving unequal access to the two sides of the prochiral carbonyl carbon atom (Fig. 3d). As mentioned above, we deduce from the differences in interaction energy that during the imprinting the majority of the MA molecules is oriented with the hydrophilic part towards the surfactant column so that the metal grows around the aromatic moiety. Thus, after removal of the template, the deeper part of the formed cavity should constitute the preferential adsorption site for the aromatic ring of PGA, allowing the carbonyl group to face the opening towards the mesopore channel. The surrounding asymmetric environment will then direct the two hydrogens, which have to be added, in such a way that the final product occupies the cavity in the most favourable configuration. This should lead to a biased stereoselective reaction of the starting compound and direct the synthesis towards the preferential formation of the enantiomer corresponding to the imprinted one (Fig. 3e).

Before using the electrodes for the electrosynthesis it is crucial to make sure that all the template molecules have left the porous structure. We have followed the progress of the template removal by cyclic voltammetry and ultraviolet–visible spectroscopy to rule out the possibility that the observed enantiomeric excess is due to a leaking of residual MA from the mesopores (Supplementary Figs 4 and 5).

**Figure 4:**
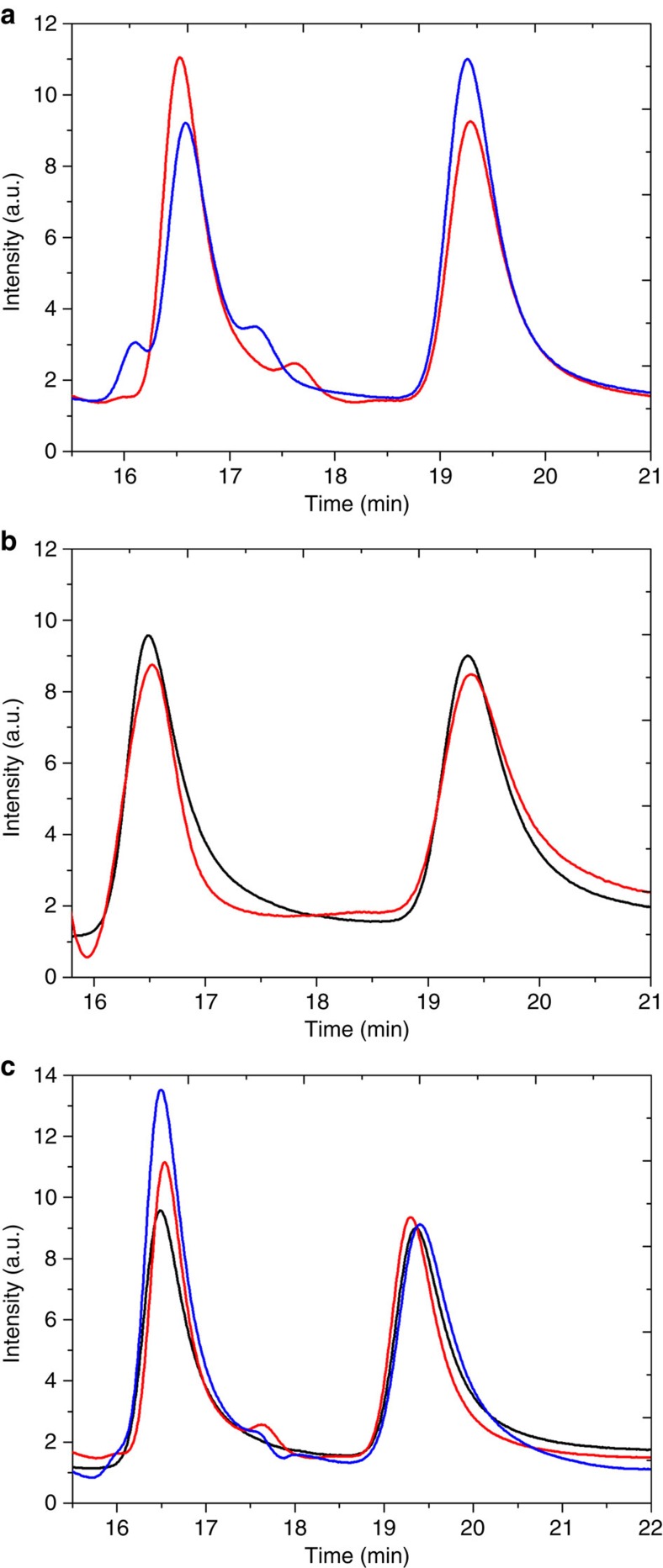
Monitoring of enantioselectivity by chromatography. HPLC chromatograms of the electrosynthesis products obtained by (**a**) an electrode imprinted with (*R*)-MA (red) or (*S*)-MA (blue) using a MA/H_2_PtCl_6_ wt ratio of 0.03. (**b**) An electrode that has been imprinted with a commercial racemic mixture of (*R*)- and (*S*)-MA (black) and an electrode that has been initially imprinted with (*S*)-MA, but for which the chiral information has been deleted by scanning the potential between −0.2 and +1.25 V in 0.5 M H_2_SO_4_ (red). (**c**) An electrode imprinted with (*R*)-MA using a (*R*)-MA/H_2_PtCl_6_ wt ratio of 0.03 (red) and 0.05 (blue), compared with the analysis of a commercial racemic mixture (black).

The global yield of the reaction obviously depends on the reaction time, the size of the electrode and the potential applied. In a typical experiment (−0.5 V, 5 h) a maximum theoretical yield of 28% can be expected (Supplementary Discussion). The degree of asymmetric electrochemical reduction of PGA using chiral-imprinted mesoporous platinum was determined by high-performance liquid chromatography (HPLC) as shown in Fig. 4, and the results of the ratio of the MA enantiomers are summarized in Table 1. The chromatograms show (*R*)- and (*S*)-MA enantiomers at retention times of about 16.5 and 19.4 min, respectively. In addition, a few small peaks were also observed in the chromatogram, indicating that a small amount of side products is formed during the reaction. As can be seen in Fig. 4a, the electrochemical reduction of PGA using (*R*)-MA-imprinted electrodes preferentially produces (*R*)-MA, illustrated by the fact that the (*R*)-MA peak is higher than the (*S*)-MA peak (red line) with a % enantiomeric excess (% ee) *o*f +5.66. The opposite is true for the (*S*)-MA-imprinted electrode (blue line), which produces in this case (*S*)-MA with a % ee of −11.73. Theoretically, the two *ee* values should be mirror values for reasons of symmetry. The fact that they differ might be explained by varying efficiencies of the imprinting process. However, the fact that they have opposite signs demonstrates that such an approach is able to favour the synthesis of one or the other enantiomer, whereas, for example, reduction by microorganisms gives access to only one of both enantiomers^43^. The enantioselectivity of the developped electrodes is even maintained if several experiments are carried out with the same electrode (Supplementary Fig. 6 and Supplementary Table 1). We typically used one electrode for three consecutive electrosynthesis runs and the obtained *ee* values are the resulting average.

In a control experiment, using the racemic mixture of commercial MA as imprinting agent (black line in Fig. 4b, and Table 1), a % ee of −1.01 is obtained (Supplementary equation (1)). Taking into account the s.e.m. of the *ee* determination (±1.13%, Supplementary equation (2)), this means that there is, as expected, no enantioselectivity in this case. Another control experiment consists in destroying the chiral nature of the electrode by electrooxidizing the platinum surface in sulfuric acid at quite positive potentials (cycling up to +1.2 V). The % ee of the resulting product obtained by electrochemical reduction on an electrode with destroyed chirality was found to be −1.52 (red line in Fig. 4b, and Table 1) indicating that the electrode has, as expected, almost completely lost its ability to induce chirality.

The efficiency of the chiral induction should theoretically depend on the number or density of imprinted cavities. To check this hypothesis, the amount of MA in the electroplating mixture has been increased. As can be seen in Fig. 4c, the peak of (*R*)-MA obtained by an electrode imprinted with (*R*)-MA using a (*R*)-MA/PtCl_6_^2−^ wt ratio of 0.05 (blue line) is significantly higher than the one obtained for an electrode synthesized with a (*R*)-MA/PtCl_6_^2−^ wt ratio of 0.03 (red line). This increase of enantioselectivity was also found for electrodes imprinted with (*S*)-MA. A % ee of −11.73 and −19.05 is observed for electrodes imprinted with (*S*)-MA/PtCl_6_^2−^ wt ratios of 0.03 and 0.05, respectively. Therefore, the number of chiral recognition sites can be tuned by varying the amount of MA in the electroplating mixture. It is tempting to try to improve the % ee by further increasing the ratio of chiral template molecules in the plating mixture. However, when the concentration of template molecules is increased too much, the phase diagram of the lyotropic liquid crystal phase is disturbed with the consequence that no more mesopores are generated. Instead a rather brittle metal deposit is obtained, which shows no significant chiral character.

## Discussion

Mesoporous platinum with imprinted cavities retaining the structural information of a chiral molecule (MA) was successfully synthesized by electrodeposition of platinum in the simultaneous presence of a lyotropic crystal phase (Brij 56) and the chiral template molecule (MA). By calculating the interaction energies between MA and Brij 56 it was found that the MA preferentially interacts with the hydrophilic part of the Brij 56 surfactant column by its –COOH group, leading to a lower energy of −12.2 kcal mol^−1^ compared with the situation where it interacts via its aromatic group. Therefore, it is reasonable to suppose that the aromatic group of MA is engulfed by the growing platinum during the electrodeposition process (Supplementary Fig. 7). The reported observations demonstrate that such double-templated matrices are able to induce the preferential formation of a given enantiomer by electroreduction of the corresponding prochiral compound. When the prochiral starting compound (PGA) is adsorbed in the chiral cavities decorating the walls of the mesopores, the present footprints provide a locally asymmetric reaction space. This allows a discrimination of the two sides of the prochiral carbon atom with respect to the spatial configuration of the initially imprinted molecule. The electrochemical reduction of PGA in (*R*)-imprinted cavities produces preferentially (*R*)-MA over (*S*)-MA enantiomer and vice versa for the (*S*)-imprinted cavities. Furthermore, the enantioselectivity of the electrodes can be amplified by increasing the number of imprinted sites in the mesoporous matrix, which allows optimizing their performance.

A major point of this work is to exclude that the observed chirality is generated by some parasitic effects, for example, due to chiral template molecules that remain entrapped in the mesoporous metal phase. It might be possible to have still adsorbed molecules even after extensive washing, which would lead to a kind of chirally modified surface rather than an encoded metal. Washing overnight demonstrated that no more molecules are desorbing (Supplementary Fig. 5 and Supplementary Discussion). However, even if no more chiral template is detected in the washing solution, this does not mean that nothing is remaining on the surface. Some molecules might still be bound and would never be detected in solution. To rule out this possibility we performed several electrosynthesis experiments using the same electrode. In all cases a significant enantiomeric excess can be observed (Supplementary Table 1 and Supplementary Discussion), making it very unlikely that this is triggered by a few physisorbed chiral molecules left over in the mesopores, because they should easily be able to desorb during such a prolonged series of electrochemical experiments.

The presented approach thus opens up interesting perspectives for the development of new materials in the context of heterogeneous chiral synthesis, and future work will be focused on generalizing this approach, either by using other metals as an imprinting matrix or by transforming other prochiral molecules into enantiomers with a high added value.

## Methods

### Chemicals

All chemicals were purchased from Sigma-Aldrich and used without further purification. MilliQ water was obtained by a Millipore system (resistivity: 18.2 MΩ cm) and used to prepare all aqueous solutions and also for all rinsing procedures. Hexachloroplatinic acid hydrate (H_2_PtCl_6_. *x*H_2_O), polyoxyethylene (10) cetyl ether (Brij 56) and MilliQ water were used to prepare the liquid crystal plating mixtures. (*R*)- and (*S*)-MA enantiomers were the chiral template molecules. PGA was used as the prochiral starting compound for the electrochemical reduction reaction. Acetic acid and sodium acetate were used to prepare acetate buffer (pH 4) solutions.

### Synthesis and characterization of chiral-imprinted mesoporous platinum

Chiral-imprinted mesoporous platinum was prepared following a literature procedure^37^, but using gold-coated glass slides with a larger size (1 cm^2^) as substrates. The gold-coated glass slides were cleaned with piranha solution, washed with MilliQ water and dried with a N_2_ stream before electrodeposition. Then, the cleaned electrodes were exposed to the liquid crystal plating mixtures, composed of 42 wt% of nonionic surfactant (Brij 56), 29 wt% of chloroplatinic acid, 29 wt% of MilliQ water and the desired amount of (*R*)- or (*S*)-MA. The electrodeposition was performed at −0.1 V, with an injected charge density of 2 C cm^−2^. After the deposition process, the electrodes were rinsed and soaked in MilliQ water overnight to remove all template molecules. The progress of the rinsing procedure can be checked by cyclic voltammetry and ultraviolet–visible spectroscopy to make sure that no more template molecules are present in the pores (Supplementary Figs 4 and 5).

Scanning electron microscopy was carried out on a Hitachi TM-1000 tabletop microscope to study the surface morphology and the thickness of the platinum films. The mesoporous morphology was investigated by transmission electron microscopy (TEM; JEOL JEM-2010). For TEM measurements, the chiral-imprinted mesoporous platinum films on Au-coated glass slides were exposed to an aqueous solution of 4 wt% KI and 1 wt% I_2_ for 20 min to dissolve the underlying Au layer. The Pt film can then be easily removed from the electrode and floated on the water surface after slow immersion of the samples into deionized water. The freestanding films were then transferred onto TEM grids.

The electroactive surface area of chiral-imprinted mesoporous platinum was estimated by measuring the integral of the hydrogen adsorption and desorption peaks in the cyclic voltammograms performed on an Autolab PGSTAT12. The cyclic voltammograms were recorded in a cell containing 0.5 M H_2_SO_4_ as electrolyte between −0.25 and +1.25 V at a scan rate of 100 mV s^−1^ (20 cycles). A calibration factor of 210 μC cm^−2^ was used to calculate the surface area of the platinum electrodes^44^. The measurement of the active surface area has been performed after the use of the electrodes for the electrosynthesis, because otherwise their chiral character would have been ereased when scanning the potential to too positive values (Fig. 4b).

### Enantioselective synthesis of MA

The electrochemical reduction of PGA on chiral-imprinted mesoporous platinum was performed for 5 h at −0.5 V versus Ag/AgCl in acetate buffer pH 4 solution containing 50 mM PGA. A three-electrode system was used with Ag/AgCl (sat. KCl), Pt mesh and the prepared mesoporous surfaces as reference, counter and working electrodes, respectively. Before the analysis of the ratio of MA enantiomers, the non-reacted PGA molecules were separated from the product mixture by using a precolumn. Then, the product mixture was evaporated and re-dissolved in mobile phase with a composition of 95% heptane, 5% *i*-propanol and 0.1% trifluoroacetic acid for analysis by HPLC. All analyses were performed on a Hitachi Chromaster analytical HPLC system equipped with a HPLC pump (5160 pump), an ultraviolet detector (5410 UV detector) and a chiral HPLC column (CHIRALPAK IC, 250 mm × 4.6 mm inner diameter) at a flow rate of 1.0 ml min^−1^. The peak fitting function of Chromaster software was used to measure the individual areas of eventually overlapping peaks in the chromatogram (Supplementary Fig. 8).

### Data availability

All relevant data that support the current findings and which are not included in the main manuscript or the Supplementary Information are available from the corresponding author on request.

## Additional information

**How to cite this article**: Yutthalekha, T. *et al*. Asymmetric synthesis using chiral-encoded metal. *Nat. Commun.* 7:12678 doi: 10.1038/ncomms12678 (2016).

## Supplementary Material

Supplementary Information Supplementary Figures 1-8, Supplementary Table 1, Supplementary Discussion and Supplementary References

## Figures and Tables

**Table 1:** Enantioselective synthesis of MA by chiral-encoded mesoporous platinum.

**Electrodes**	Ratio of imprinted molecule*	**% Enantiomeric excess**†
(*R*)-MA imprinted	0.03	5.66
	0.05	10.71
(*S*)-MA imprinted	0.03	−11.73
	0.05	−19.05
Destroyed chirality	—	−1.52
Commercial racemic mixture	—	−1.01

^*^Weight ratio of MA to H_2_PtCl_6_ in the electroplating mixture.

^†^% Enantiomeric excess (% ee)=(RMA−SMA)/(RMA+SMA) × 100, where RMA and SMA are the HPLC peak areas of (*R*)-MA and (*S*)-MA, respectively. The calculated s.e.m. is ±1.13% ee (Supplementary equation (2)).
