# Supplementary material for: Asymmetric synthesis using chiral-encoded metal
Source: Nat Commun. 2016 Aug 26;7:12678. doi: 10.1038/ncomms12678 (PMC5007459; doi:10.1038/ncomms12678)
Supplement: Supplementary Information — Supplementary Figures 1-8, Supplementary Table 1, Supplementary Discussion and Supplementary References [file ncomms12678-s1.pdf]

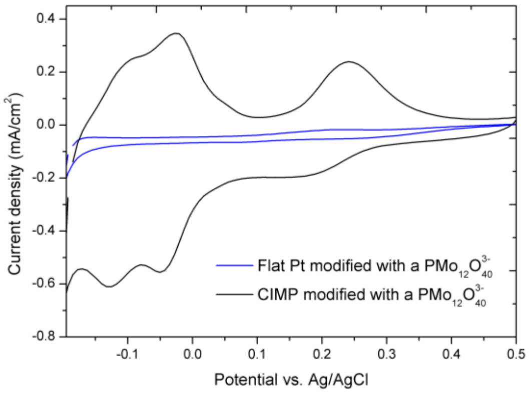

Supplementary Figure 1 CV signal of a monolayer of $PMo_{12}O_{40}^{3-}$ on flat (blue) and mesoporous (black) platinum. Both electrodes have been dipped in a 5mM aqueous solution of $H_3PMo_{12}O_{40}$ for one hour and then the signal is measured in pure supporting electrolyte (0.5M H_2SO_4).

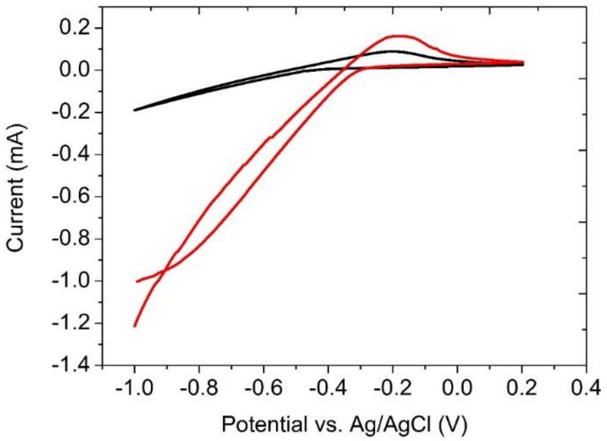

Supplementary Figure 2 Cyclic voltammograms of a normal (non-porous) platinum electrode in acetate buffer (pH4) (black) and in acetate buffer containing 50 mM PGA (red) at a scan rate of

100 mV s^{-1}, showing that the reduction of the prochiral C=O functional group in the PGA molecule starts around -0.3 V.

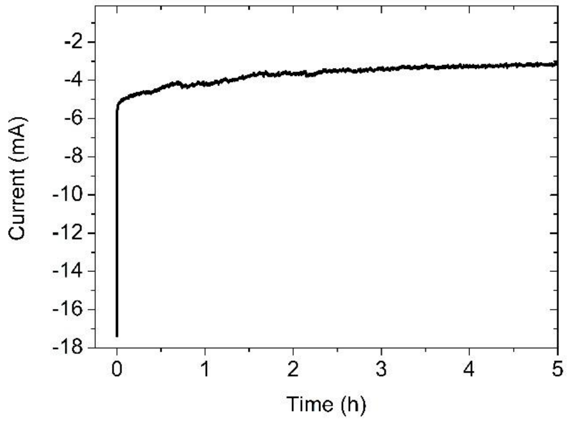

Supplementary Figure 3 Chronoamperometric curve during a typical electroreduction experiment in a 50mM solution of PGA when a potential of -0.5V is applied to the working electrode.

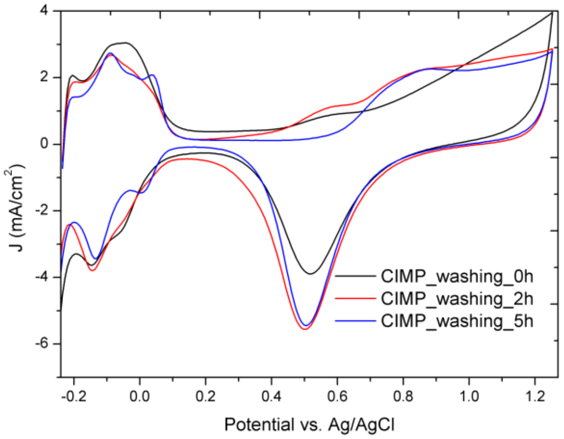

Supplementary Figure 4 Cyclic voltammograms (scan rate 100 mV s^{-1}) of a chiral imprinted mesoporous electrode in 0.5 M H_2SO_4 after washing for different durations in MilliQ water. The

parasitic redox signal in the range of 0.2-0.6 V, present for short rinsing, disappears when washing for a longer time.

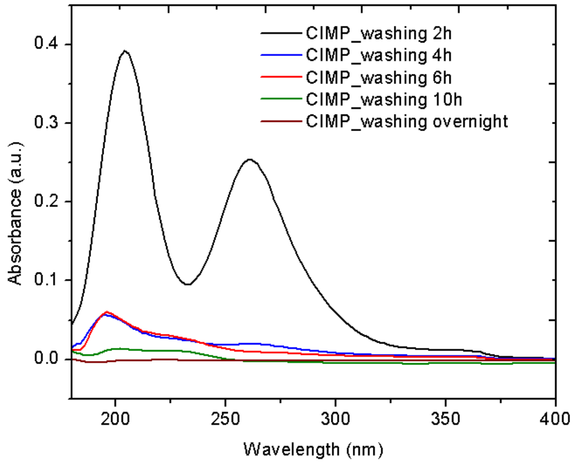

Supplementary Figure 5 Series of UV-Vis spectra recorded in the different washing solutions that are used for the template removal.

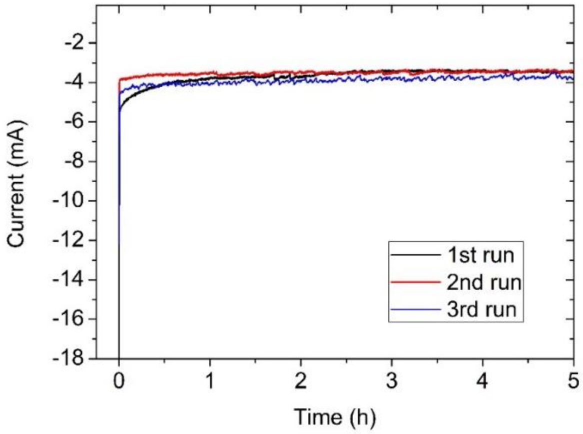

Supplementary Figure 6 Chronoamperometric curves of three consecutive electroreduction experiments with the same imprinted electrode in a 50 mM solution of PGA when a potential of -

0.5V is applied to the working electrode. The data were obtained with an electrode imprinted with (*R*)-MA/H_2PtCl_6 wt. ratio of 0.03.

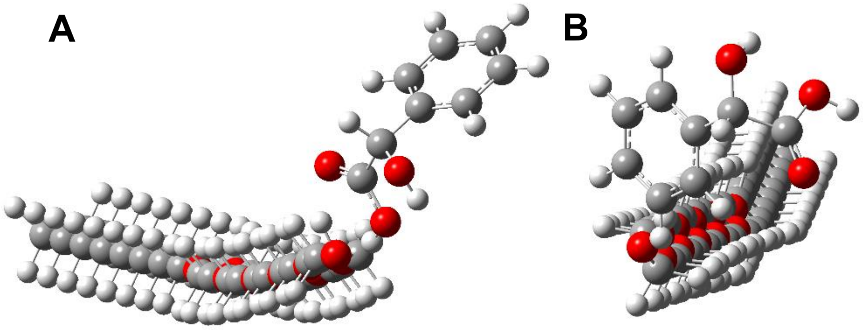

Supplementary Figure 7 Optimized structures representing A) the interaction of Brij 56 and mandelic acid *via* the –COOH group and B) the interaction of Brij 56 and mandelic acid *via* the aromatic group.

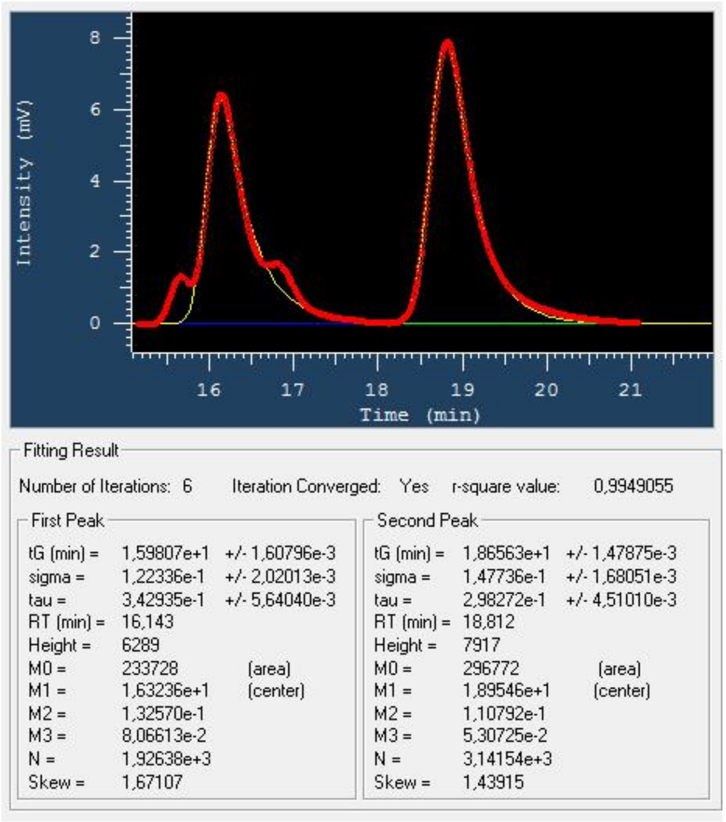

Supplementary Figure 8 Fitting results (yellow) of overlapping peaks in the HPLC chromatogram obtained with Chromaster software.

Supplementary Table 1 Enantioselective synthesis of mandelic acid using the same imprinted electrode (The data were taken from the electrode imprinted with a MA/H_2PtCl_6 wt. ratio of 0.05)

**(*R*)-imprinted electrode**	**%*ee***
1st Run	+13.01
2nd Run	+10.83
3rd Run	+8.30

Supplementary Discussion

Choice of electrode potential for the electrosynthesis

In order to perform the electroreduction of phenyglyoxylic acid (PGA) into madelic acid a sufficiently negative potential has to be applied to the working electrode. The onset of the reduction current has been evaluated by cyclic voltammetry in a solution containing acetate buffer and the prochiral molecule. As can be seen from Supplementary Figure 2, significant reduction appears for potentials more negative than -0.3 V when a classic platinum electrode is used (red curve). Therefore a constant potential of -0.5 V has been chosen for all reduction experiments as a compromise between a sufficiently fast electrochemical conversion of PGA, and limited interference with hydrogen evolution, which starts around -0.5 V as can be seen from

the black curve. The currents that are typically obtained under these conditions when using a mesoporous electrode are in the range of a few mA (see Supplementary Figure 3).

Removal of the template molecules from the mesoporous electrode

Once the electrodeposition of platinum has been carried out in the presence of the lyotropic liquid crystal and a given enantiomer of mandelic acid, both template molecules have to be carfully removed from the mesoporous structure in order to liberate the pores and to ensure that chiral induction is not originating from left-over enantiomers in the pores. As both molecules are water soluble the most efficient way to eliminate them is exposure of the electrode to MilliQ water for prolonged time. The progress of washing can be monitored by cyclic voltammetry, as illustrated in Supplementary Figure 4. For relatively short times of rinsing, the CV recorded in sulfuric acid does not show the classic signal for platinum (like in Figure 2b of the main manuscript), but several parasitic peaks are present in the potential range from 0.2 V to 0.6 V (Supplementary Figure 4 black curve), resulting from the oxidation of template molecules, which are still present in the mesopores. One can also see from the signal in the hydrogen adsorption region (-0.2 to +0.1V) that the surface is "polluted". After two hours of washing (red curve) the situation improves and the characteristic features of a platinum CV become more pronounced, but the oxidation peak at +0.55 V indicates that there is still template present in the pores. After five hours of washing the situation further improves and an almost ideal CV is obtained (blue curve). As a consequence, all electrodes used for the electrosynthesis have been cleaned by washing for at least 12 hours.

An additional way to study the gradual release of template molecules from the mesoporous structure is to monitor their concentration in the different washing solutions by performing UV-Vis spectroscopic measurements. Right after the plating the mesoporous platinum was immersed into 50 ml of pure water for two hours. Then the spectrum was measured (see Supplementary Figure 5, black curve). The electrode was then again transferred into pure water for another two hours, which resulted in the blue curve, already indicating a much smaller release of molecules. In the next two washing steps decreasing amounts of molecules are released (red and green

curve). When the electrode is kept overnight in fresh water, no more mandelic acid can be detected in the washing solution. These observations already give a quite strong indication that the measured enantiomeric excess can't be due to a leaking of residual mandelic acid from the mesopores.

An even stronger argument, ruling out an eventual pollution of the synthesis solution with chiral molecules left over in the electrode from the imprining process, is the fact that one and the same imprinted electrode can be used several times in a row to perform electrosynthesis resulting in significant enantiomerci excess. The details are described in the next paragraph.

Electrosynthesis using the same imprinted electrode

Electrosynthesis of mandelic acid is performed with the same imprinted electrode following the same conditions (5h electrolysis of 20 ml of a 50 mM PGA solution at -0.5V). After each electrosynthesis experiment, the used electrode was soaked in MilliQ water overnight to remove all adsorbed compounds. Then, the same electrode was used for the subsequent experiments. As can be seen in Supplementary Figure 6, the electrode doesn't undergo major degradation, such as passivation or fouling, as the chronoamperometric curves for three consecutive runs of the electrode are quite similar.

The enantiomeric excess obtained from three consecutive experiments with the same electrode is summarized in Supplementary Table 1. The (*R*)-imprinted electrode gave products with the corresponding handedness in all experiments. The observed gradual decrease of ee is most likely due to the fact that slowly the Pt surface is losing its chiral character due to a reorganization of the metal atoms.

These control experiments demonstrate that chiral induction can be obtained not only for the first use, but also in the subsequent experiments. This again indicates that the observed ee cannot be the effect of chiral molecules left over in the mesopores after the washing process.

Calculation of global yield and measurement of enantiomeric excess by HPLC

The electrosynthesis is carried out at a constant potential of -0.5 V and in a typical experiment an average current of 3mA is flowing through the cell. If we assume in a first order approximation a faradic efficiency of 100% (a reasonable value taking into account that the current due to proton reduction is still very small at this potential, see black curve in Supplementary Figure 2) then we can estimate the maximum global yield of the reaction for a given reaction time. Under the present conditions (5h electrolysis of 20ml of a 50mM solution at -0.5V with an average current of 3mA) the yield is 28%. This value can be increased by prolonging the time of the electrolysis, the macroscopic size of the electrode and eventually by going to more negative potentials.

After the electrosynthesis, unreacted PGA is separated from the product mixture by using a precolumn (CHIRALPAK IA). The collected sample is then evaporated and re-dissolved in a mobile phase (95% heptane, 5% *i*-propanol and 0.1% trifluoroacetic acid) for analysis by high-performance liquid chromatography (HPLC) with a CHIRALPAK IC column. The integration of the area under the peaks has been carried out with Chrommaster software. In the case of overlapping peaks like in the chromatogram illustrated in Supplementary Figure 8, the software allowed also to deconvolute the contribution of different species to the global signal. The estimated areas have been used to calculate the enantiomeric excess (% *ee*) of two enantiomers via supplementary equation 1 (in the present case of an electrode imprinted with (S)-mandelic acid, a % *ee* of -11.88 is obtained). Moreover, three independent electrosynthesis experiments

with the same imprinted electrode were performed to calculate the standard error of the mean via supplementary equation 2. A value of ±1.13 % *ee* was obtained.

$$\% \boldsymbol{ee} = \frac{(\boldsymbol{RMA}-\boldsymbol{SMA})}{(\boldsymbol{RMA}+\boldsymbol{SMA})}\ \boldsymbol{x100} \qquad (1)$$

where RMA and SMA are HPLC peak areas of (*R*)-mandelic acid and (*S*)-mandelic acid, respectively.

$$\boldsymbol{SEM} = \frac{\boldsymbol{S}}{\sqrt{\boldsymbol{n}}} \qquad (2)$$

where S is the standard deviation and n is the number of experiments.

Calculation of interaction energy difference

To get further information about the interaction between Brij 56 and mandelic acid, the interaction energies of two different orientations of mandelic acid (*via* its –COOH group and aromatic group) and Brij 56 (as shown in Supplementary Figure 7), were calculated. All structure optimizations were carried out using the Gaussian 03 program with a B3LYP hybrid functional and a 6-31G(d,p) basis set[1]. It was found that the mandelic acid preferentially interacts with the hydrophilic part of the Brij 56 surfactant by its –COOH group, leading to a lower energy of -12.2 kcal/mol compared to the situation where it interacts *via* its aromatic group. Therefore it is reasonable to suppose that the aromatic group of mandelic acid is engulfed by the growing platinum during the electrodeposition process. This should favor during the subsequent asymmetric electrosynthesis step the adsorption of the aromatic ring of PGA in the deeper part of the cavity, allowing the carbonyl group to face the opening towards the mesopore channel.

Porosity evaluation by chemisorption of polyoxometalates

It is interesting to evaluate the porosity also with other, eventually bigger, molecules diffusing from the outside, because due to its small size hydrogen adsorption will only give the maximum value of surface area. This can be done for example by looking at the adsorption of polyoxometalates under electrochemically relevant conditions. These molecules are much

bigger (ca. 1nm diameter) and therefore allow to estimate the accessible porosity even for such large entities. We've used this surface chemistry based on literature reports to evaluate in an independent way the porosity[2]. The Supplementary Figure 1 illustrates the redox signal of adsorbed $PMo_{12}O_{40}^{3-}$ anions inside the pores (black line) compared to a signal obtained with a flat platinum electrode (blue line). The signal measured for the flat electrode is almost not visible at the current scale necessary for the porous electrode. Integration of the electric charge involved in the redox process allows to conclude that the accessible internal surface for molecules of this size is still a factor 30 higher compared to a flat electrode.

Supplementary References

1 Frisch, M. J. et al, Gaussian 03, revision B.05 ed., Gaussian, Inc. Pittsburgh, PA, 2003.

2 Kulesza, P. et al. Network films composed of conducting polymer-linked and polyoxometalate-stabilized platinum nanoparticles. *Chemistry of Materials* **16**, 4128-4134 (2004).
